# Aberrant IL-21/STAT3 signaling disrupts regulatory T cell function and CD4^+^ T cell homeostasis in children with type 1 diabetes

**DOI:** 10.3389/fimmu.2026.1791993

**Published:** 2026-05-18

**Authors:** Xiaofeng Sun, Jiou Zhao, Jin Liu, Wendi Zhou

**Affiliations:** Department of Pediatrics, The Affiliated Huaian No.1 People’s Hospital of Nanjing Medical University, Huaian, Jiangsu, China

**Keywords:** autoimmune immunity, CD4^+^ T cells, IL-21/STAT3 signaling, regulatory T cells, T cell homeostasis, type 1 diabetes

## Abstract

**Background:**

Type 1 diabetes (T1D) is an autoimmune disease characterized by profound dysregulation of CD4^+^ T cell subsets, particularly impaired regulatory T cell (Treg) function accompanied by excessive Th17 and T follicular helper (Tfh) cell responses. Interleukin-21 (IL-21) has been implicated in T cell–mediated autoimmunity; however, the immunoregulatory mechanisms linking IL-21 signaling to T cell imbalance in pediatric T1D remain incompletely understood.

**Methods:**

Single-cell RNA sequencing data from children with T1D were analyzed to characterize IL-21/STAT3 pathway activity across immune cell subsets. Functional assays were performed using primary human CD4^+^ T cells and Tregs treated with IL-21 and the STAT3 inhibitor Stattic. T cell differentiation, suppressive function, cytokine production, and STAT3 activation were assessed *in vitro*. The immunological and pathological effects of STAT3 inhibition were further evaluated in a non-obese diabetic (NOD) mouse model.

**Results:**

Single-cell transcriptomic analysis revealed enhanced IL-21/STAT3 signaling activity in CD4^+^ T cell populations from children with T1D. IL-21 stimulation induced STAT3 phosphorylation and nuclear translocation, leading to reduced FoxP3 expression, impaired Treg-associated suppressive function, and a shift in CD4^+^ T cell differentiation toward Th17 and Tfh phenotypes. Pharmacological inhibition of STAT3 effectively reversed IL-21–mediated Treg dysfunction and restored CD4^+^ T cell balance *in vitro*. In NOD mice, STAT3 inhibition ameliorated hyperglycemia, reduced pancreatic inflammation, preserved insulin-positive islets, and corrected systemic T cell subset imbalance.

**Conclusion:**

Aberrant activation of the IL-21/STAT3 signaling axis may contribute to impaired Treg function and CD4^+^ T cell imbalance in pediatric T1D. These findings suggest that IL-21–STAT3–dependent immune dysregulation represents an important mechanism involved in T cell imbalance and autoimmune pathology in T1D.

## Introduction

Type 1 diabetes (T1D) is a chronic autoimmune disorder arising from the interaction of genetic predisposition and environmental influences, ultimately resulting in immune-mediated destruction of pancreatic β cells (1, 2). Among children and adolescents, T1D represents one of the most common endocrine and metabolic disorders. It is estimated that more than one million children and adolescents worldwide are affected by T1D, characterized by early onset and a lifelong disease course, imposing substantial medical, economic, and psychological burdens on patients and their families (3, 4). Alarmingly, the global incidence of T1D is increasing at an annual rate of approximately 3%, posing an escalating public health challenge (5). The burden of T1D extends far beyond the need for lifelong insulin replacement therapy; chronic hyperglycemia progressively damages both microvascular and macrovascular systems, resulting in severe complications such as retinopathy, nephropathy, neuropathy, and cardiovascular disorders, which remain the primary causes of disability and mortality among affected individuals (6). It is widely accepted that T1D originates from a breakdown of immune tolerance in genetically predisposed individuals; however, the precise immunological cascade driving the transition from subclinical autoimmunity to overt hyperglycemia has yet to be fully elucidated.

Mounting evidence supports the notion that adaptive immune responses—particularly CD4^+^ T cell–dependent cellular immunity—are key contributors to the pathogenesis of T1D (7). Under physiological conditions, immune tolerance to self-antigens is maintained through tightly regulated mechanisms, among which regulatory T cells (Tregs) function as critical “brakes” within the immune system. Tregs suppress the activation and expansion of autoreactive T cells through contact-dependent interactions (e.g., CTLA-4) and the release of immunosuppressive cytokines including interleukin-10 (IL-10) and transforming growth factor-β (TGF-β), thereby preserving peripheral immune tolerance (8, 9). In contrast, both patients with T1D and experimental animal models exhibit reduced Treg frequency and/or functional impairment, leading to a marked decline in immunosuppressive capacity and failure to restrain autoimmune responses (10, 11). Concurrently, pro-inflammatory T helper 17 (Th17) cells and T follicular helper (Tfh) cells are aberrantly expanded. Th17-derived cytokines, including IL-17, promote the recruitment of neutrophils and other inflammatory cells, exacerbating tissue injury, whereas Tfh cells facilitate autoreactive B-cell activation and autoantibody generation, jointly contributing to pancreatic islet destruction (12–14). Thus, the imbalance characterized by impaired Treg function and enhanced Th17/Tfh effector activity is widely regarded as a pivotal event driving β-cell demise. Identifying the upstream signaling pathways that orchestrate this T-cell subset disequilibrium remains a critical objective for understanding T1D pathogenesis and developing effective therapeutic strategies.

Among the candidate signaling molecules implicated in T-cell dysregulation, interleukin-21 (IL-21) and its downstream signal transducer and activator of transcription 3 (STAT3) pathway have attracted increasing attention in recent years. Activated CD4^+^ T cells, especially Tfh cells, represent the major source of IL-21, with additional contributions from natural killer T (NKT) cells (15, 16). Genome-wide association studies (GWAS) have revealed single-nucleotide polymorphisms (SNPs) in the IL-21 gene locus that are significantly linked to genetic susceptibility to T1D (17). Moreover, genetic ablation of IL-21 or its receptor effectively prevents diabetes development in the non-obese diabetic (NOD) mouse model (18, 19). These compelling genetic and experimental findings highlight IL-21 as a key pathogenic mediator in T1D. Mechanistically, IL-21 signals primarily through activation of STAT3 within the JAK–STAT pathway (20). STAT3 is a well-established master transcription factor governing the differentiation of Th17 and Tfh cells (21). Notably, emerging evidence suggests that under inflammatory conditions, excessive STAT3 activation may interfere with STAT5-dependent signaling required for Treg stability and function, thereby promoting Treg dysfunction (22). In light of these observations, we hypothesize that the IL-21/STAT3 signaling axis represents a critical upstream pathway in T1D that simultaneously promotes Th17/Tfh differentiation while impairing regulatory T-cell function, thereby resulting in immune imbalance and β-cell destruction.

## Materials and methods

### Single-cell RNA sequencing data acquisition and preprocessing

Single-cell RNA sequencing (scRNA-seq) data were retrieved from the Gene Expression Omnibus (GEO) database under accession number GSE221297. This dataset consists of scRNA-seq profile from 12 human peripheral blood samples, including nine pediatric patients with T1D and three healthy controls. The demographic and clinical characteristics of these subjects were extracted from GSE221297 and are summarized in **Table 1**. Quality control (QC) was performed on the raw gene expression matrices of each sample using the Python package Scanpy (v1.9.0). Cells expressing fewer than 200 genes were excluded, and genes detected in fewer than three cells were filtered out. Cells passing QC were normalized for sequencing depth, and genes with high variability were identified. Dimensionality reduction was next conducted via principal component analysis (PCA), followed by construction of a k-nearest-neighbor graph based on the PCA results. Cell clustering was conducted using the Leiden algorithm, and Uniform Manifold Approximation and Projection (UMAP) was employed for nonlinear dimensionality reduction and global cellular landscape visualization.

**Table 1:** Demographic and clinical characteristics of pediatric patients with type 1 diabetes and healthy controls (derived from GSE221297).

Variables	Healthy donors (n=3)	T1D (n=9)
Age (years)	7.0 ± 2.6	7.9 ± 2.5
Sex (M/F)	2/1	5/4
BMI z-score	-0.08 ± 0.56	-0.12 ± 1.14
Disease duration (years)	NA	1.44 ± 0.86
Fasting C-peptide (pmol/L)	177.7 ± 11.8	158.4 ± 64.0
Stimulated C-peptide (pmol/L)	659.3 ± 204.2	352.0 ± 205.0
Insulin dose (U/kg/day)	NA	0.29 ± 0.22
HbA1c (%)	5.2 ± 0.0	9.0 ± 2.1
GADA positive (n)	0	6
IA2A positive (n)	0	7
ZnT8A positive (n)	0	8

### Cell type annotation

To identify cell types within the resulting clusters, the automated cell annotation tool CellTypist (v1.0) was applied using the built-in immune reference dataset “Immune_All_Low.pkl.” This machine learning–based approach assigns each cell to the most probable immune cell type by comparing its gene expression profile with reference signatures. Following annotation, the proportions of immune cell subsets in each sample were calculated, and a sample-by–cell type heatmap was generated to visualize immune-cell composition differences between T1D patients and healthy controls.

### Gene set construction and pathway activity scoring

Two pathway-related gene sets were obtained from the Molecular Signatures Database (MSigDB; v2025.1.Hs): (1) REACTOME_INTERLEUKIN_21_SIGNALING, comprising genes such as IL21, IL21R, IL2RG, JAK1, JAK3, STAT1, STAT3, STAT4, STAT5A, and STAT5B; (2) KEGG_JAK_STAT_SIGNALING_PATHWAY.

Single-cell–level pathway activity was quantified using the R package AUCell (v1.20.0). Briefly, AUCell ranks all genes within each cell according to expression levels, generating a ranked gene list for each cell. The enrichment of a given gene set within this ranking is then evaluated by calculating the area under the recovery curve (Area Under the Curve, AUC). Higher AUC values indicate greater activity of the corresponding signaling pathway within individual cells.

### Differential analysis and visualization

To compare pathway activity between the T1D and control groups within specific immune-cell subsets, pathway AUC scores were extracted from key populations, including T follicular helper (Tfh/Tfh-17) cells and regulatory T (Treg) cells. Between-group differences were assessed using the R-based Wilcoxon rank-sum test (wilcox.test()), with a significance threshold of P < 0.05.

In addition, the expression patterns of key components of the IL-21/STAT3 axis (IL21, IL21R, and STAT3) were visualized on UMAP embeddings to examine their distribution across immune-cell subsets and to compare expression profiles between T1D and control samples.

### Cell model establishment and treatments

Peripheral blood mononuclear cells (PBMCs) were obtained from healthy donor peripheral blood (10–20 mL) through Ficoll density gradient centrifugation. Using an untouched isolation kit, CD4^+^ T cells were sorted from PBMCs by fluorescence-activated cell sorting (FACS). To further obtain distinct T-cell subsets, CD4^+^CD25^+^ regulatory T cells (Tregs) were purified from the CD4^+^ T-cell fraction using a CD4^+^CD25^+^ Treg-specific isolation kit, while CD3^+^CD4^+^CD25^−^ (FoxP3^−^) effector T cells (Teff) were obtained by depleting CD25^+^ cells. All cells were grown in RPMI-1640 complete medium supplemented with 10% fetal bovine serum (FBS), penicillin (100 U/mL), and streptomycin (100 μg/mL) at 37 °C in a humidified 5% CO_2_ incubator. The CD4^+^ T cells were assigned to the following experimental groups: (1) NC group: cells without any treatment; (2) IL−21 group: cells exposed to IL−21 (100 ng/mL) for 48 hours; (3) IL−6 group: cells stimulated with IL−6 (50 ng/mL) for 48 hours. The Treg cells were allocated into the following experimental groups: (1) NC group: cells without any treatment; (2) IL−21 group: cells incubated with IL−21 (100 ng/mL) for 48 hours; (3) Stattic group: cells treated with the STAT3 inhibitor Stattic (2 μmol/L) alone; (4) IL−21 + Stattic group: cells pre−incubated with Stattic (2 μmol/L) for 1 hour, followed by co−treatment with IL−21 (100 ng/mL) for a total of 48 hours.

### Treg–Teff co-culture assay

Freshly isolated Treg cells were cultured together with carboxyfluorescein succinimidyl ester (CFSE)–labeled Teff cells at a 1:1 ratio. Cells were seeded into 96-well U-bottom plates at 1 × 10^5^ cells per well and cultured in RPMI-1640 medium supplemented with 10% FBS, penicillin (100 U/mL), and streptomycin (100 μg/mL). The co-culture system was assigned to the following groups: (1) Teff group (positive control): only Teff cells (1×10^5^ cells/well) were seeded without Treg cells; (2) NC group: co−culture of Treg and Teff cells without any drug treatment; (3) IL-21 group: co-culture treated with IL-21 (100 ng/mL); (4) Stattic group: the STAT3 inhibitor Stattic (2 μmol/L) was added to the co−culture system; (5) IL-21 + Stattic group: co-culture pretreated with Stattic (2 μmol/L) for 1 h followed by IL-21 (100 ng/mL). All co-culture experiments were performed at 37 °C with 5% CO_2_ for the indicated durations prior to downstream analyses.

### Animal model

Female NOD mice aged 6–12 weeks were used in this study (n = 6 animals in each group) and stratified into the following groups: (1) Control group: C57BL/6 mice were included as negative controls and received the same volume of DMSO; (2) NOD-Early group: 8-week-old NOD mice with blood glucose levels <11.1 mmol/L and treated with the same volume of DMSO; (3) NOD-Onset group: 12-week-old NOD mice characterized by sustained hyperglycemia (>11.1 mmol/L for three consecutive days) and treated with the same volume of DMSO; (4) NOD + Stattic group: NOD mice at disease onset treated with Stattic (50 mg/kg) via oral gavage on alternate days for two weeks. All animal experiments were conducted at the Guangdong Medical Laboratory Animal Center, which provides certified experimental facilities and animal housing conditions. Animals were maintained in a specific pathogen-free facility with controlled environmental conditions (22 ± 2 °C, 50% ± 10% humidity), a 12-h light/dark cycle, and ad libitum access to food and water. All experimental procedures complied with institutional guidelines and were approved by the Animal Ethics Committee. A schematic overview of the experimental design and treatment timeline is provided in **Supplementary Figure 1**.

### Flow cytometry analysis

Cells from each experimental group were collected, washed, and resuspended in prechilled flow cytometry staining buffer. For murine spleen samples, spleens were mechanically dissociated under sterile conditions and filtered using a 70-μm cell strainer to yield single-cell suspensions. Erythrocytes were lysed using red blood cell lysis buffer, followed by two washes with phosphate-buffered saline (PBS). After cell counting, staining was performed using 1 × 10^6^ cells. Surface-marker staining was performed by incubating cells with fluorochrome-conjugated antibodies against mouse CD3 (100236, 1:200, BioLegend, USA), CD4 (100406, 1:200, BioLegend, USA), CD25 (102008, 1:200, BioLegend, USA), and other indicated markers for 30 min at 4 °C while protected from light. Cells were washed twice following incubation to remove unbound antibodies. For intracellular staining of FoxP3 (45-5773-82, 1:100, eBioscience, USA) and IL-17A (12-7177-81, 1:100, eBioscience, USA), cells were processed for fixation and permeabilization with a FoxP3 transcription factor staining buffer set (00-5523-00, eBioscience, USA) according to the manufacturer’s guidelines, followed by incubation with the corresponding antibodies at 4 °C for 45–60 min with protection from light. After staining, cells were resuspended in staining buffer, passed through a cell strainer, and immediately subjected to flow cytometric analysis (FACSDiva™, BD FACSymphony A5, BD Biosciences, USA). The proportions of CD4^+^CD25^+^FoxP3^+^ Treg cells, CD3^+^CD4^+^CD25^−^FoxP3^−^ Teff cells, Th17 cells (CD4^+^IL-17A^+^), CD4^+^CXCR5^+^PD-1^+^ T follicular helper (Tfh) cells, and the expression level of CTLA-4 were quantified.

### Western blot analysis

Cells and splenic tissues were harvested and lysed using RIPA buffer (20115ES60, Yeasen, China) to extract total protein. Protein concentrations were determined using a bicinchoninic acid (BCA) assay kit (20201ES86, Yeasen, China). Equal amounts of protein (typically 20–40 μg) were mixed with 5× SDS-PAGE loading buffer and denatured at 95 °C for 5 min in a heat block. Proteins were separated by SDS–PAGE and transferred onto polyvinylidene difluoride (PVDF) membranes (1620177, Bio-Rad, USA). Primary antibody incubation was performed on membranes using antibodies against STAT3 (ab68153, 1:1000, Abcam, UK), phosphorylated STAT3 (p-STAT3; ab76315, 1:1000, Abcam, UK), Bcl-6 (ab241549, 1:1000, Abcam, UK), FoxP3 (ab215206, 1:1000, Abcam, UK), and β-actin (4970, 1:1000, Cell Signaling Technology, USA), before incubation with horseradish peroxidase (HRP)–linked secondary antibodies: goat anti-rabbit IgG (H+L) (7074, 1:1000, CST, USA) or goat anti-mouse IgG (H+L) (7076, 1:1000, CST, USA). Protein bands were detected by enhanced chemiluminescence (ECL) (P0018S, Beyotime, China), acquired using a ChemiDoc™ XRS+ imaging system (Bio-Rad, USA), and measured using ImageJ software. Protein expression levels were adjusted using β-actin as an internal control.

### Immunofluorescence staining

After the indicated treatments, cells were washed using prechilled phosphate-buffered saline (PBS, pH 7.4) followed by fixation with 4% paraformaldehyde for 15 min at room temperature. Cells were treated with PBS containing 0.1% Triton X-100 to achieve permeabilization at room temperature for 10 min. Thereafter, cells were incubated overnight at 4 °C within a humidified chamber with a rabbit monoclonal antibody against phosphorylated STAT3 (p-STAT3; 9145, 1:200, CST, USA) diluted in antibody diluent consisting of PBS supplemented with 1% bovine serum albumin. Cells were washed with PBS and subsequently incubated with Alexa Fluor 488–conjugated, cross-adsorbed goat anti-rabbit IgG (H+L) secondary antibodies (A-11008 or A-11001, 1:500, Invitrogen, USA) at room temperature for 1 h under dark conditions. DAPI (1 μg/mL) was used to counterstain nuclei for 10 min at room temperature while protected from light. Fluorescence microscopy was conducted with a confocal laser scanning microscope (LSM980, ZEISS, Germany). p-STAT3 signals were captured with excitation at 488 nm and emission collected between 500 and 550 nm (green fluorescence), whereas DAPI signals were captured using 405 nm excitation and 410–480 nm emission (blue fluorescence). ImageJ software (NIH, USA) was used to quantitatively analyze p-STAT3 fluorescence intensity.

### Enzyme-linked immunosorbent assay

Supernatants from cell cultures and peripheral blood serum samples from mice were obtained following the indicated treatments. The concentrations of IL-10 (E-EL-H6154, Elabscience, China), TGF-β (E-EL-H1587, Elabscience, China), and IL-21 (E-EL-H0106, Elabscience, China) were measured using commercial ELISA kits in accordance with the manufacturers’ guidelines. Measurements of absorbance were performed at 450 nm using a fully automated microplate reader (Multiskan™ FC, Thermo Fisher Scientific, USA), with 630 nm used as the reference wavelength. Standard curves were generated by plotting cytokine standard concentrations against their corresponding optical density (OD) values and fitting the data using a four-parameter logistic (4-PL) regression model. Cytokine concentrations in samples were calculated automatically based on the standard curve equations.

### CFSE proliferation assay

Teffs during exponential-phase growth were collected, resuspended in prewarmed PBS, and standardized to a concentration of 1 × 10^7^ cells/mL. An equal volume of cell suspension was mixed with 5 μM carboxyfluorescein succinimidyl ester (CFSE; C34554, Thermo Fisher Scientific, USA) to achieve a working concentration of 2.5 μM, with subsequent incubation at 37 °C for 20 min while protected from light. Staining was terminated by adding a fivefold volume of ice-cold complete medium containing 10% FBS. Cells were pelleted at 300 × g for 5 min at 4 °C and subjected to three consecutive washes with prechilled PBS. CFSE-labeled Teff cells were cultured together with unlabeled Treg cells at a 1:1 ratio (5 × 10^4^ cells each) in 96-well U-bottom plates with a final volume of 200 μL per well. Experimental grouping and treatments were identical to those described in the co-culture experiments. Cells were kept at 37 °C with 5% CO_2_ for a duration of 96 h. After co-culture, cells were harvested and washed prior to analysis by flow cytometry on a FACSymphony A5 system (BD Biosciences, USA). CFSE fluorescence was excited at 488 nm and detected in the FITC channel to assess Teff cell proliferation.

### Blood glucose monitoring

During the experimental period, blood glucose levels of mice in each group were measured every 3 days at a fixed time point (between 9:00 and 11:00 a.m.). Prior to measurement, mice underwent a 6-h fasting period with ad libitum access to water. blood samples were harvested via tail-tip puncture with a disposable lancet, and glucose levels were measured with a glucometer (OneTouch UltraVue, Johnson & Johnson, USA) with compatible test strips. Blood glucose values were recorded directly and expressed in mmol/L.

### Hematoxylin and eosin staining and insulitis scoring

Upon completion of the experiment, mice were sacrificed, and pancreatic tissues were immediately harvested, fixed with 4% paraformaldehyde, embedded in paraffin, sectioned, and processed for H&E staining. Islet morphology and inflammatory infiltration were examined by light microscopy, and insulitis was graded based on previously established criteria (23): score 0, no infiltration; score 1, lymphocytes infiltrating <25% of the islet area; score 2, 25–50% islet involvement; score 3, >50% islet involvement. Each section contributed at least 20 randomly selected islets that were independently evaluated by two blinded pathologists. The average score was calculated and used as the final insulitis score for each sample.

### Immunohistochemistry

Immunohistochemical staining was performed using a commercial IHC detection kit (RK05872, ABclonal, China). Paraffin-embedded pancreatic sections were deparaffinized, rehydrated, and subjected to heat-induced antigen retrieval. Endogenous peroxidase activity was quenched by incubation with 3% hydrogen peroxide for 10 min. Sections were then blocked with normal goat serum at room temperature for 30 min. Without washing, sections were incubated overnight at 4°C under humidified conditions with a mouse monoclonal antibody against insulin (3014, 1:200, Cell Signaling Technology, USA). Following PBS washing, sections were incubated with a species-appropriate biotinylated secondary antibody at room temperature for 30 min, after which horseradish peroxidase–conjugated streptavidin was applied for 30 min. Signal development was achieved using diaminobenzidine (DAB) substrate, with reaction times (typically 1–5 min) monitored under a microscope. Hematoxylin counterstaining was performed on sections prior to dehydration, clearing, and mounting. Immunoreactive insulin-positive areas appeared as brown or dark-brown staining localized in the cytoplasm of pancreatic β cells. Semi-quantitative analysis of insulin-positive area or integrated optical density was conducted through Image-Pro Plus software.

### Statistical analysis

All statistical analyses related to bioinformatic data were performed using R software (v4.2.0). Differences in pathway activity scores between groups were assessed using the nonparametric Wilcoxon rank-sum test. Statistical significance was established at a P value < 0.05. GraphPad Prism 9.0 (GraphPad Software, San Diego, CA, USA) was used for statistical analysis. Values are reported as mean ± standard deviation (SD). Normality and homogeneity of variance were assessed by the Shapiro–Wilk test and Levene’s test, respectively. When data met the assumptions of normality and equal variance, group differences were analyzed using one-way analysis of variance (ANOVA) followed by Tukey’s *post hoc* test. For repeated measurements at different time points (e.g., blood glucose monitoring), repeated-measures ANOVA was applied, with Bonferroni-adjusted multiple comparisons. Statistical significance was assumed for P values < 0.05.

## Results

### Single-cell RNA sequencing analysis reveals enhanced IL-21/JAK pathway activity in immune cells of patients with T1D

**Figure 1:**
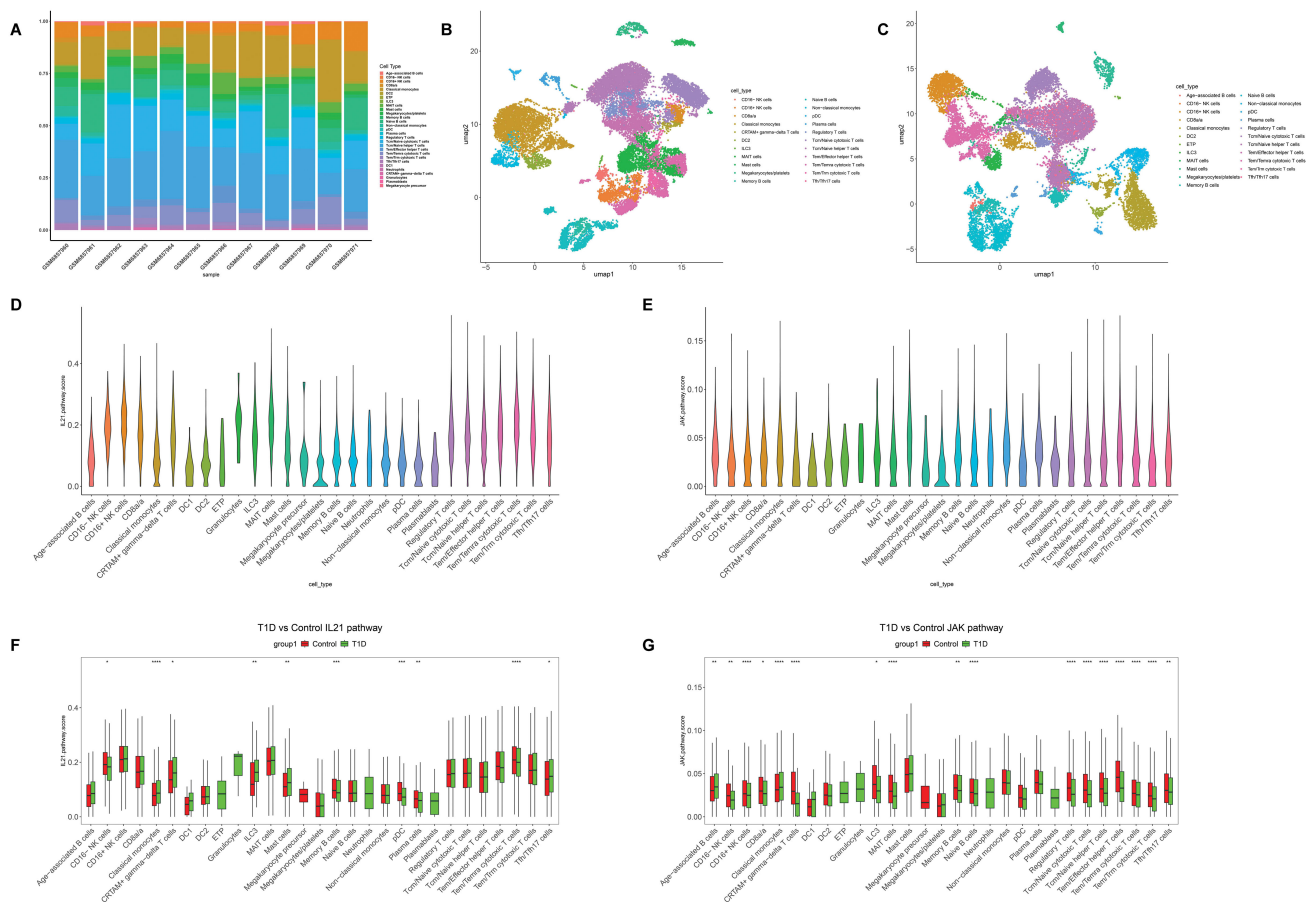
Analysis of single-cell RNA sequencing data delineates the expression features of the IL-21/JAK signaling network in immune cells from patients with type 1 diabetes (T1D). **(A)** Proportional distribution of major immune-cell subsets across individual T1D and control samples in the GSE221297 dataset. **(B)** Immune cell clustering visualized using UMAP from a representative healthy control sample (GSM6857966). **(C)** Clustering patterns of immune cells visualized by UMAP from a representative T1D sample (GSM6857960). **(D)** Violin plots showing AUCell scores of the IL-21 signaling pathway across different immune-cell subsets. **(E)** Violin plots showing AUCell scores of the JAK signaling pathway across different immune-cell subsets. **(F)** Comparative analysis of IL-21 pathway activity scores between patients with T1D and healthy controls. **(G)** Comparison of JAK pathway activity scores between the T1D and control groups. Statistical comparisons between the T1D and control groups were performed using the Wilcoxon rank-sum test. *P < 0.05, **P < 0.01, ***P < 0.001, and ****P < 0.0001.

To systematically characterize alterations in key signaling pathways within the immune microenvironment of patients with T1D at the single-cell level, we undertook an extensive analysis of the public scRNA-seq dataset GSE221297, which includes samples from patients with T1D and healthy controls. Initial analysis of immune-cell composition revealed that T cells constituted the predominant immune population across all samples. Compared with healthy controls, several T1D samples exhibited altered proportions of specific immune subsets, including regulatory T cells and plasma cells, indicating pronounced heterogeneity in peripheral immune-cell composition among patients with T1D (**Figure 1A**).

To visualize the global distribution of immune-cell populations, Uniform Manifold Approximation and Projection (UMAP)–based clustering was performed. In a representative healthy control sample (GSM6857966; **Figure 1B**), distinct immune-cell populations—such as CD16^−^ monocytes, plasmacytoid dendritic cells (pDCs), classical monocytes, and multiple T- and B-cell subsets—formed well-separated and clearly defined clusters in UMAP space. In contrast, representative T1D samples displayed a tendency toward partial overlap between T-cell and B-cell subsets, although major immune-cell lineages remained identifiable. This altered distribution pattern suggests increased immune-cell activation or state transitions in patients with T1D (**Figure 1C**).

To further investigate signaling pathway activity across immune-cell subsets, pathway activity scores for the IL-21 and JAK signaling cascades were calculated using the AUCell algorithm. Both pathways exhibited relatively high activity scores in multiple T- and B-cell subsets, including T follicular helper (Tfh)/Tfh17 cells, memory B cells, and plasma cells, indicating that these populations may represent primary responders to IL-21/JAK signaling (**Figures 1D, E**).

Next, we directly compared pathway activity between the T1D and control groups within specific immune-cell subsets. Compared with healthy controls, patients with T1D showed significant differences in the activity of both the IL-21 pathway (**Figure 1F**) and the JAK pathway (**Figure 1G**) across several key immune populations. Notably, activated memory B cells and CD4^+^ memory T cells from the T1D group exhibited markedly higher median pathway activity scores, accompanied by a broader distribution range, reflecting enhanced intercellular variability.

To further substantiate the potential activation of IL-21/STAT3–mediated signaling in T1D, we visualized the expression patterns of IL21, IL21R, and STAT3 on UMAP embeddings derived from scRNA-seq data (**Supplementary Figure 1**). These analyses suggested that STAT3 expression and IL-21 pathway activity were preferentially enriched within specific CD4^+^ T-cell populations, namely Tfh, Th17, and Treg cells. Collectively, these findings indicate that IL-21/STAT3–mediated signaling may be activated within the immune microenvironment of T1D and predominantly affects distinct CD4^+^ T-cell subsets. Based on these observations, we subsequently conducted functional experiments to validate the immunoregulatory role of the IL-21/STAT3 axis at both the expression and functional levels.

### Sorting and flow cytometric validation of CD4^+^ T-cell subsets (Treg and Teff)

**Figure 2:**
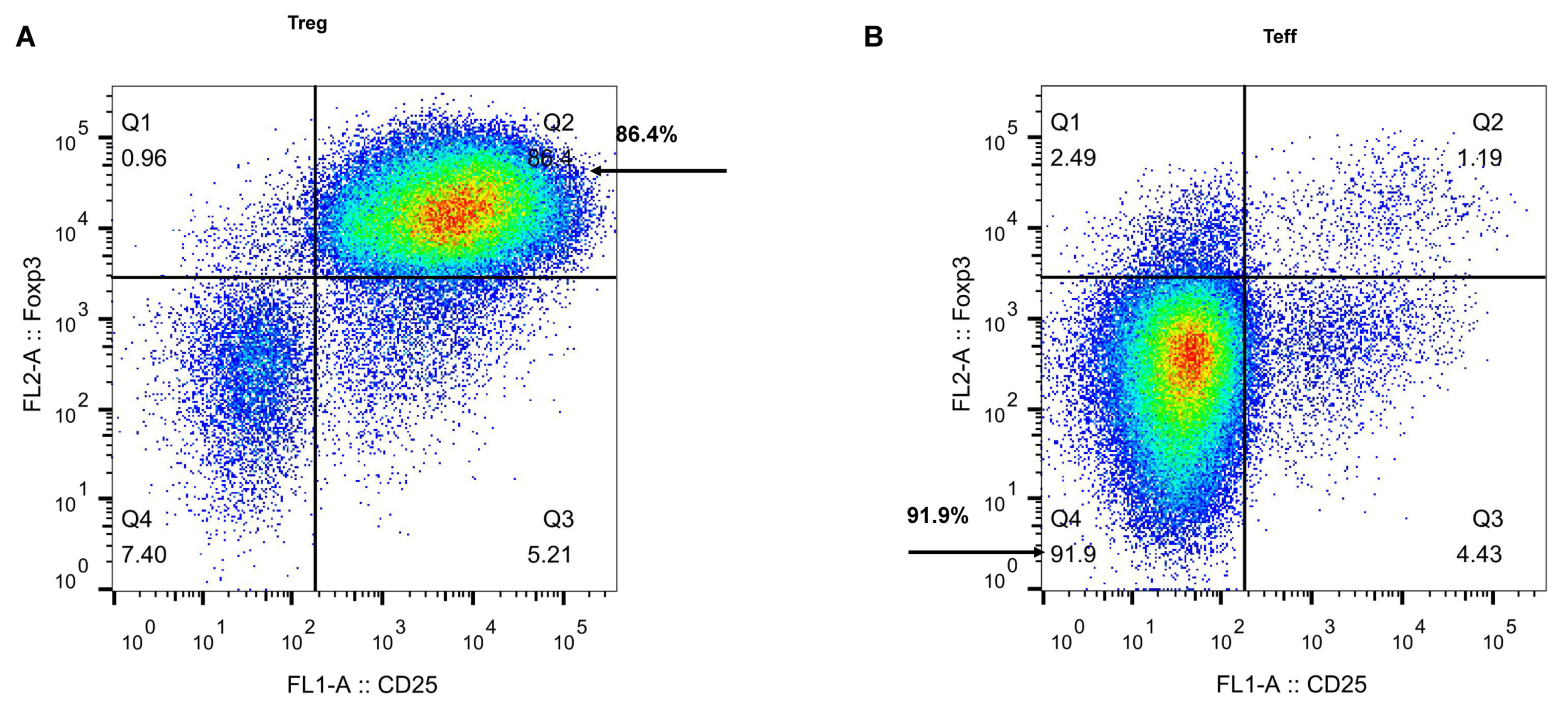
Sorting and flow cytometric validation of CD4^+^ T-cell subsets (Treg and Teff). **(A)** Flow cytometric assessment of the purity of CD4^+^CD25^+^FoxP3^+^ regulatory T cells (Tregs). **(B)** Flow cytometric assessment of the purity of CD3^+^CD4^+^CD25^−^FoxP3^−^ effector T cells (Teffs).

To ensure the reliability of subsequent functional assays, PBMCs were first isolated from healthy donors, and flow cytometric cell sorting was performed to specifically obtain CD4^+^CD25^+^FoxP3^+^ Tregs and CD3^+^CD4^+^CD25^−^FoxP3^−^ Teffs. Immediately after sorting, the purity of both cell populations was assessed by flow cytometry. Analysis of the sorted Treg population demonstrated that more than 85% of cells were CD4^+^CD25^+^FoxP3^+^ triple-positive, indicating successful enrichment of a highly purified Treg population (**Figure 2A**). Similarly, purity assessment of the sorted Teff population showed that over 90% of cells were CD3^+^CD4^+^CD25^−^FoxP3^−^, with effective depletion of FoxP3^+^ cells, confirming the acquisition of a Teff population of sufficient purity (**Figure 2B**).

Collectively, the cumulative evidence indicates that the sorting strategy employed in the present study framework efficiently and specifically isolated highly purified Treg and Teff cell populations, thereby providing a robust cellular model for subsequent investigations into the contribution of the IL-21/STAT3 signaling cascade to Treg function.

### Cytokine stimulation induces STAT3 activation and alters CD4^+^ T-cell subset distribution

**Figure 3:**
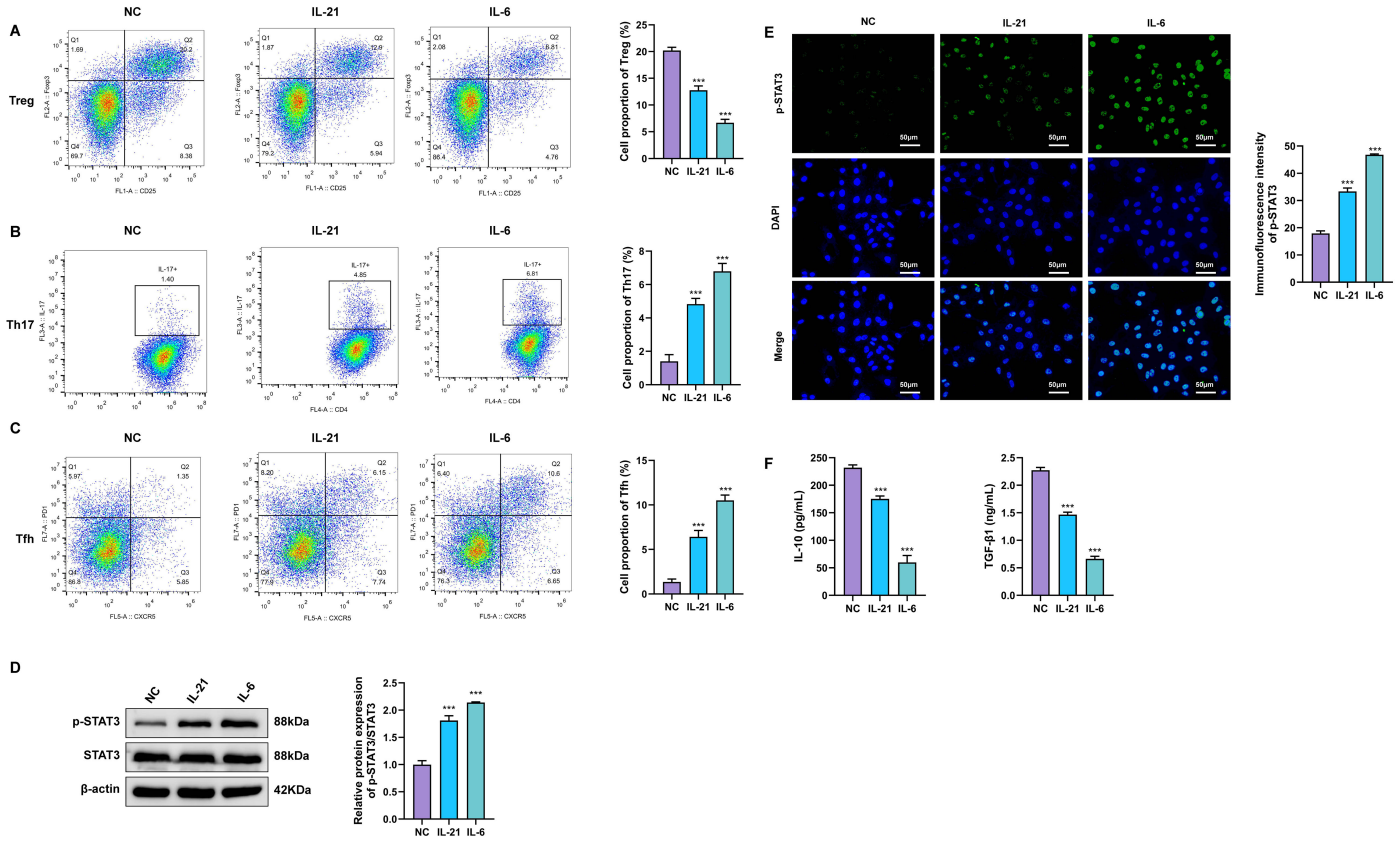
Cytokine stimulation induces STAT3 activation and alters CD4^+^ T-cell subset distribution. ^(A–C)^ Flow cytometric analysis of the proportions of Tregs **(A)**, Th17 cells **(B)**, and T follicular helper (Tfh) cells **(C)** among CD4^+^ T cells in the NC, IL-21, and IL-6 groups. **(D)** Expression of total STAT3 and phosphorylated STAT3 (p-STAT3) in CD4^+^ T cells assessed by Western blot following IL-21 or IL-6 stimulation **(E)** Immunofluorescence (IF) staining showing nuclear translocation of p-STAT3 in CD4^+^ T cells from the NC, IL-21, and IL-6 groups. **(F)** Enzyme-linked immunosorbent assay (ELISA) measuring the levels of IL-10 and TGF-β in culture supernatants from the NC, IL-21, and IL-6 groups. ***P < 0.001 vs. NC group.

To investigate how IL-21 and IL-6 modulate STAT3 signaling and immunoregulatory functions in CD4^+^ T cells, purified CD4^+^ T cells were subjected to IL-21 (100 ng/mL) or IL-6 (50 ng/mL) stimulation for 48 h. Analysis by flow cytometry showed that, when compared with the untreated control group (NC), IL-21 stimulation significantly altered CD4^+^ T-cell subset distribution. Specifically, CD4^+^CD25^+^FoxP3^+^ regulatory T cells (Tregs) exhibited a marked reduction in proportion, whereas CD4^+^IL-17^+^ Th17 cells and CD4^+^CXCR5^+^PD-1^+^ T follicular helper (Tfh) cells were significantly increased. Notably, IL-6 exerted a more pronounced effect on these subsets, leading to a further decrease in Treg frequency and a greater expansion of Th17 and Tfh cells, suggesting that IL-6 may be more potent than IL-21 in driving CD4^+^ T-cell differentiation toward pro-inflammatory phenotypes (**Figures 3A–C**).

Western blot analysis demonstrated that both IL-21 and IL-6 stimulation significantly induced phosphorylation of STAT3 at Tyr705 (p-STAT3), with the IL-6–treated group exhibiting the most robust increase in p-STAT3 levels (**Figure 3D**). Consistent with these findings, IF staining showed that in the NC group, p-STAT3 signals were predominantly localized in the cytoplasm, with minimal nuclear fluorescence. In contrast, IL-21 stimulation resulted in evident nuclear translocation of p-STAT3, concomitant with a marked elevation in nuclear fluorescence intensity. The IL-6–treated group displayed the most pronounced nuclear accumulation, with p-STAT3 signals almost exclusively localized to the nucleus (**Figure 3E**).

ELISA results further indicated that, in comparison with the NC group, IL-21 stimulation led to a significant reduction in the secretion of the immunosuppressive cytokines IL-10 and TGF-β, whereas IL-6 stimulation caused an even greater decrease in the levels of both cytokines (**Figure 3F**). Collectively, these results demonstrate that both IL-21 and IL-6 effectively activate STAT3 signaling in CD4^+^ T cells by promoting STAT3 phosphorylation and nuclear translocation, thereby driving CD4^+^ T-cell subset reprogramming.

### Stattic inhibits IL-21–induced STAT3 activation, restores the Treg phenotype, and suppresses Th17/Tfh polarization

**Figure 4:**
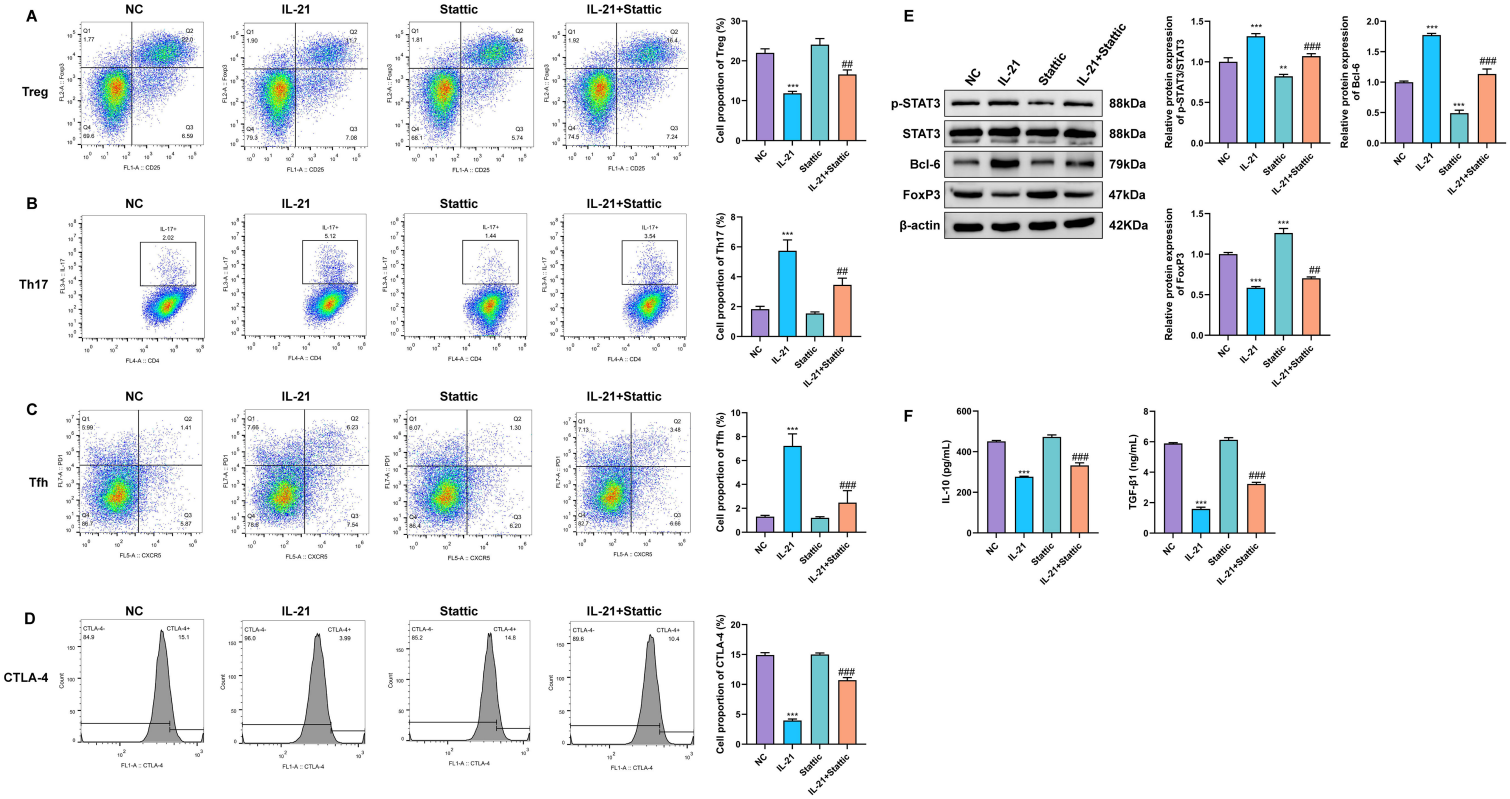
Stattic inhibits IL-21–induced STAT3 activation, restores the Treg phenotype, and suppresses Th17/Tfh polarization. **(A–C)** Flow cytometric analysis of the proportions of Tregs **(A)**, Th17 cells **(B)**, and Tfh cells **(C)** among Treg cell cultures in the NC, IL-21, Stattic, and IL-21 + Stattic groups. **(D)** Assessment of CTLA-4 expression by flow cytometry in cells from the NC, IL-21, Stattic, and IL-21 + Stattic groups. **(E)** Protein expression of STAT3, p-STAT3, Bcl-6, and FoxP3 assessed by Western blot in cells from the NC, IL-21, Stattic, and IL-21 + Stattic groups. **(F)** ELISA measuring secreted IL-10 and TGF-β levels in culture supernatants from the NC, IL-21, Stattic, and IL-21 + Stattic groups. **P < 0.01, ***P < 0.001 vs. NC group; ^##^P < 0.01, ^###^P < 0.001 vs. IL-21 group.

To verify whether IL-21–driven effects on Treg cell function is mediated through STAT3 signaling, purified Treg cells were treated with the STAT3-specific inhibitor Stattic (2 μmol/L). Flow cytometric analysis showed that treatment with Stattic alone did not induce significant alterations in the proportions of T-cell subsets relative to the untreated control group (NC). Alternatively, upon combined treatment with IL-21, Stattic effectively reversed the IL-21–induced phenotypic alterations. Specifically, compared with the IL-21–treated group, the combined treatment group exhibited a significant restoration of the Treg cell proportion, accompanied by marked reductions in Th17 and Tfh-like cell populations (**Figures 4A–C**).

In addition, analysis of the key Treg inhibitory molecule CTLA-4 revealed that IL-21 stimulation significantly downregulated CTLA-4 expression, whereas Stattic treatment alone maintained CTLA-4 expression at baseline or slightly elevated levels. Importantly, co-treatment with Stattic markedly antagonized the IL-21–induced suppression of CTLA-4, resulting in partial restoration of its expression (**Figure 4D**).

Western blot analysis further demonstrated that Stattic treatment not only reduced basal levels of phosphorylated STAT3 (p-STAT3), but also completely abrogated the IL-21–induced upregulation of p-STAT3 and Bcl-6, while concomitantly restoring the expression of FoxP3 (**Figure 4E**). Consistent with these findings, ELISA results showed that Stattic alone did not significantly affect IL-10 and TGF-β release. In contrast, co-treatment with Stattic and IL-21 significantly reversed the IL-21–mediated suppression of these immunosuppressive cytokines, leading to a marked recovery in IL-10 and TGF-β concentrations compared with IL-21 treatment alone (**Figure 4F**).

Collectively, these results demonstrate that the STAT3 inhibitor Stattic effectively blocks IL-21–mediated STAT3 activation and the associated cellular alterations, thereby confirming STAT3 as a critical molecular mediator through which IL-21 regulates Treg cell function.

### Suppressive effects of Treg cells on Teff cell proliferation and polarization

**Figure 5:**
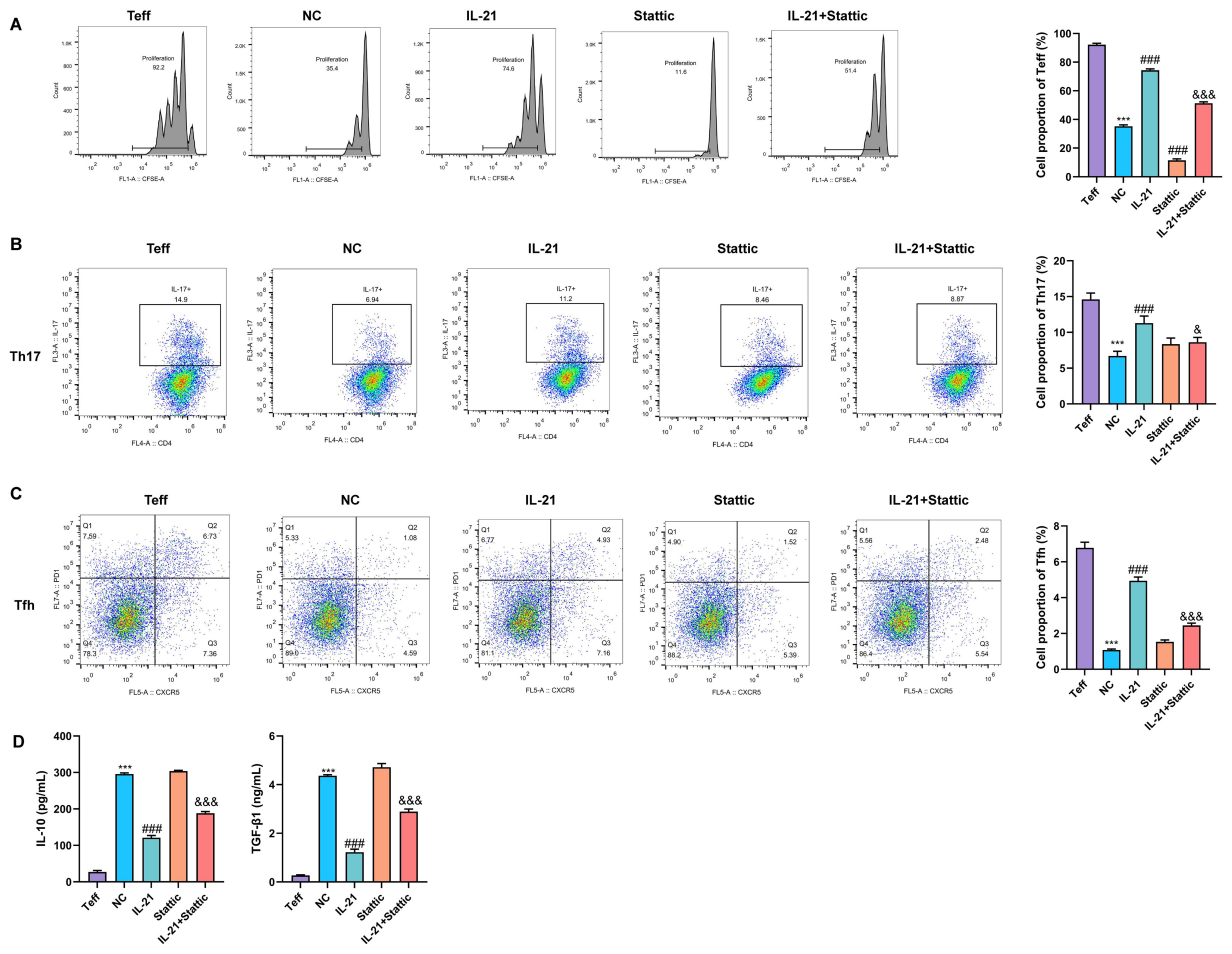
Suppressive effects of Treg cells on Teff cell proliferation and polarization. **(A)** CFSE-based proliferation assay assessing the suppressive effect of Treg cells on Teff cell proliferation. **(B, C)** Proportional analysis of Th17 **(B)** and Tfh **(C)** cells among Teff cells by flow cytometry in the co-culture system. **(D)** ELISA measuring the levels of IL-10 and TGF-β in culture supernatants from the NC, IL-21, Stattic, and IL-21 + Stattic groups. ***P < 0.001 vs. Teff group; ^###^P < 0.001 vs. NC group; ^&^P < 0.05, ^&&&^P < 0.001 vs. IL-21 group.

To determine whether the suppressive function of Treg cells is regulated by the IL-21/STAT3 signaling pathway, an *in vitro* co-culture system was constructed with Treg cells and CFSE-labeled Teffs. This system was used to explore the influence of Treg cells on Teff proliferation, polarization, and cytokine secretion. CFSE dilution analysis revealed that, in contrast to Teff cells cultured alone, co-culture with untreated Treg cells (NC group) resulted in a marked suppression of Teff proliferation, as evidenced by higher CFSE fluorescence intensity and reduced dye dilution. In contrast, the addition of IL-21 to the co-culture system significantly impaired the suppressive capacity of Treg cells, leading to enhanced CFSE dilution and restoration of Teff proliferation. Treatment with Stattic alone further strengthened the suppressive function of Treg cells, resulting in maximal inhibition of Teff proliferation. Importantly, Stattic partially reversed the negative effects of IL-21 on Treg-mediated suppression (**Figure 5A**).

Subsequent flow cytometric analysis of Teff cells following co-culture demonstrated that the proportions of Th17 and T follicular helper (Tfh) cells were highest in the Teff-only culture group. Co-culture with Treg cells effectively suppressed Teff differentiation toward Th17 and Tfh phenotypes. IL-21 treatment markedly attenuated this suppressive effect, accompanied by a significant rise in the proportions of Th17 and Tfh cells. Stattic alone exerted effects on Th17/Tfh differentiation comparable to those observed in the NC group, whereas co-treatment with Stattic and IL-21 significantly reversed the IL-21–induced increases in Th17 and Tfh cell proportions (**Figures 5B, C**).

ELISA results further showed that, compared with Teff cells cultured alone, Treg–Teff co-culture significantly led to elevated secretion of the immunosuppressive cytokines IL-10 and TGF-β. IL-21 treatment markedly suppressed the production of both cytokines. Stattic alone did not significantly affect cytokine secretion; however, it effectively counteracted the IL-21–mediated inhibitory effects on IL-10 and TGF-β production (**Figure 5D**).

Collectively, these findings demonstrate that Treg cells exert potent suppressive effects on Teff cell proliferation and their polarization toward Th17 and Tfh lineages. Moreover, the STAT3 inhibitor Stattic not only enhances the basal suppressive function of Treg cells but also effectively reverses IL-21–induced functional impairment. These results identify the IL-21/STAT3 axis as a critical regulatory hub governing Treg-mediated immunosuppression.

### STAT3 inhibition ameliorates hyperglycemia, islet injury, and immune dysregulation in T1D mice

**Figure 6:**
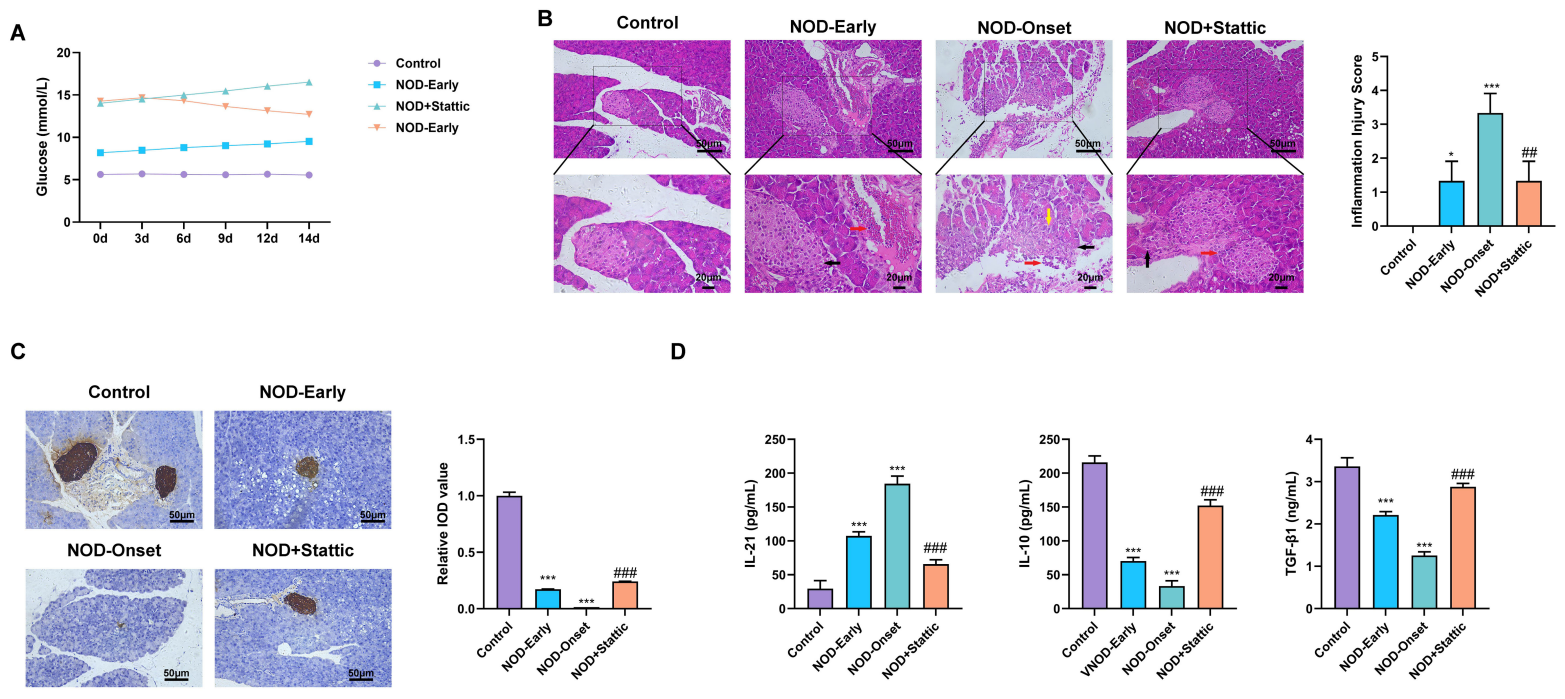
STAT3 inhibition ameliorates hyperglycemia, islet injury, and immune cytokine imbalance in T1D mice. **(A)** Glycemic levels obtained in mice from each experimental group. **(B)** Representative hematoxylin and eosin (H&E)–stained pancreatic sections showing histopathological changes, along with quantitative insulitis scores. **(C)** Immunohistochemical (IHC) analysis of insulin expression in pancreatic islets. **(D)** ELISA measuring serum levels of IL-21, IL-10, and TGF-β in mice from each group. *P < 0.05, ***P < 0.001 vs. Control group; ^##^P < 0.01, ^###^P < 0.001 vs. NOD-Onset group.

To validate the implication of STAT3 signaling in the onset and progression of T1D and to evaluate its therapeutic applicability, we assessed the effects of the STAT3 inhibitor Stattic in a spontaneous T1D NOD mouse model. Blood glucose monitoring showed that glucose levels in healthy control mice remained within the normal range throughout the experimental period, whereas mice at disease onset (NOD-Onset) exhibited persistently elevated glycemic levels. In contrast, NOD mice treated with Stattic (NOD + Stattic) displayed a marked reduction in blood glucose beginning approximately 6 days after treatment initiation, followed by stabilization at lower levels (**Figure 6A**).

Pancreatic histopathology was subsequently evaluated by hematoxylin and eosin (H&E) staining to assess insulitis. Control mice exhibited intact islet architecture with well-defined boundaries and no evident inflammatory infiltration. In early-stage NOD mice, mild lymphocytic infiltration was observed, predominantly localized to the peri-islet regions. In contrast, NOD-Onset mice displayed severe insulitis, characterized by extensive lymphocytic infiltration leading to blurred islet boundaries and substantial structural disruption. Notably, pancreatic pathology was significantly ameliorated in Stattic-treated mice, as evidenced by reduced lymphocyte infiltration, improved preservation of islet architecture, and significantly lower insulitis scores compared with untreated NOD-Onset mice (**Figure 6B**).

Immunohistochemical analysis of insulin expression further revealed robust and uniformly distributed insulin-positive staining within islets of control mice. Early-stage NOD mice showed reduced insulin staining intensity and a decreased number of insulin-positive β cells, whereas insulin expression was markedly diminished or nearly absent in NOD-Onset mice. Importantly, Stattic treatment significantly restored both the area and intensity of insulin-positive staining within pancreatic islets compared with the NOD-Onset group (**Figure 6C**).

Serum cytokine analysis by ELISA demonstrated that, relative to control mice, IL-10 and TGF-β levels progressively declined as NOD disease progressed, whereas the pro-inflammatory cytokine IL-21 was significantly elevated. Treatment with Stattic effectively reversed these alterations, restoring cytokine profiles toward an immunoregulatory state (**Figure 6D**).

Collectively, these findings indicate that in the NOD mouse model, pharmacological inhibition of STAT3 by Stattic effectively lowers blood glucose levels, attenuates islet inflammation, preserves β-cell function, and rebalances systemic immune cytokine profiles toward immune tolerance.

### The IL-21/STAT3 pathway regulates T-cell subset distribution and key molecular expression in the spleen and lymphoid tissues

**Figure 7:**
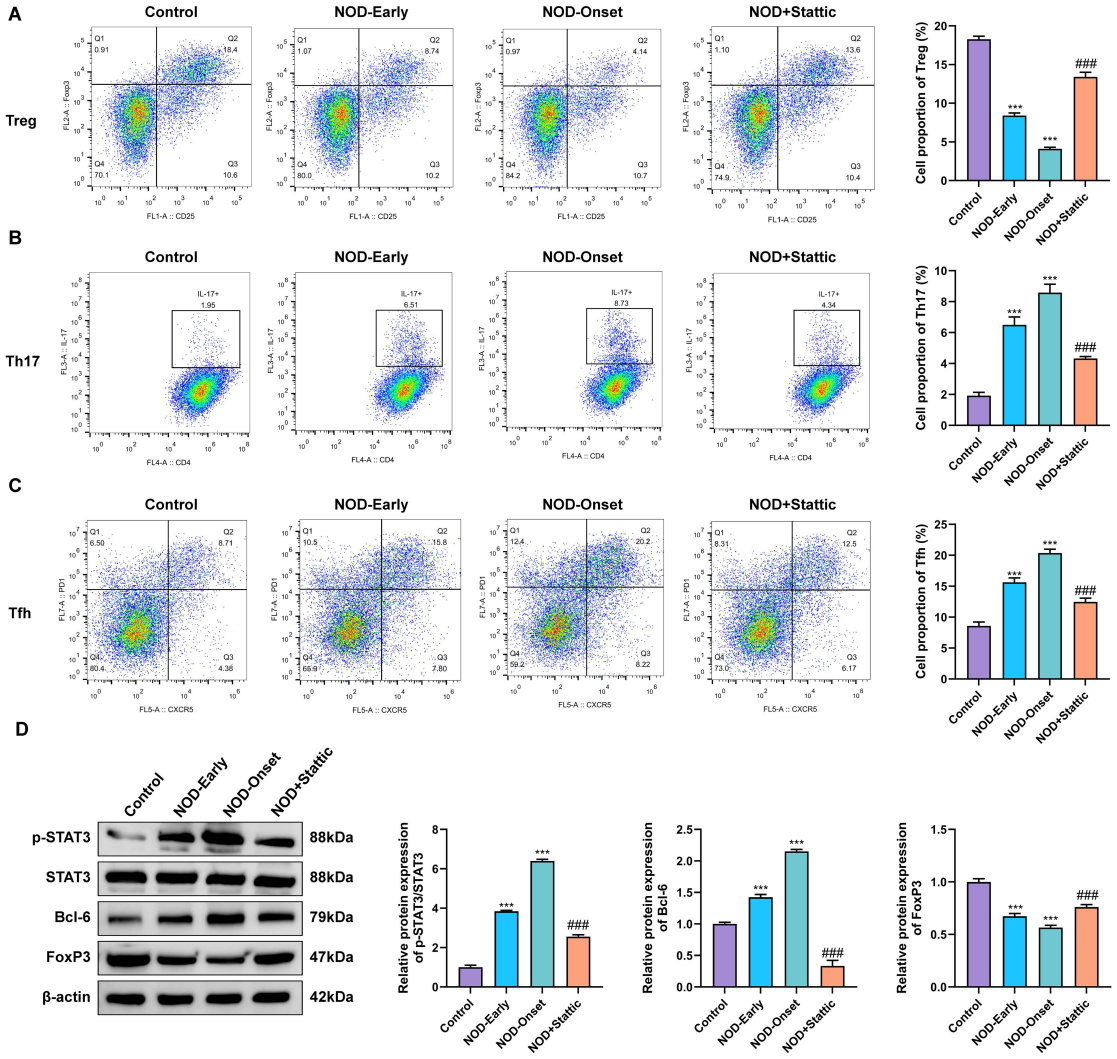
The IL-21/STAT3 pathway regulates splenic T-cell subset distribution and key molecular expression in T1D mice. **(A–C)** Assessment of the proportions of Tregs **(A)**, Th17 cells **(B)**, and Tfh cells **(C)** among splenic lymphocytes from each experimental group by flow cytometry. **(D)** Western blot detection of STAT3, p-STAT3, Bcl-6, and FoxP3 proteins in splenic tissues of each experimental group. ***P < 0.001 vs. Control group; ^###^P < 0.001 vs. NOD-Onset group.

To further delineate the contribution of the IL-21/STAT3 signaling pathway to systemic immune regulation in the T1D animal model, we investigated its effects on splenic T-cell subset balance and associated signaling molecule expression. Flow cytometric analysis revealed a marked imbalance in splenic T-cell subsets in NOD mice versus controls. Notably, treatment with the STAT3 inhibitor Stattic (NOD + Stattic) effectively corrected this imbalance in NOD mice at disease onset, as evidenced by a marked increase in the frequency of regulatory T cells (Tregs) and a concomitant decrease in Th17 and T follicular helper (Tfh) cell populations (**Figures 7A–C**).

Western blot analysis further demonstrated that, relative to control mice, NOD mice exhibited significantly elevated levels of phosphorylated STAT3 (p-STAT3) and upregulated Bcl-6 expression, a pivotal transcription factor promoting Tfh cell differentiation, whereas FoxP3 expression, the central transcription factor for Treg cells, was markedly reduced. These molecular alterations were most pronounced at the disease-onset stage. Importantly, Stattic treatment substantially suppressed the excessive expression of p-STAT3 and Bcl-6 while promoting the restoration of FoxP3 protein levels (**Figure 7D**).

Collectively, these findings indicate that *in vivo* activation of the IL-21/STAT3 pathway in T1D model mice drives splenic T-cell subset polarization toward pro-inflammatory Th17/Tfh phenotypes by upregulating p-STAT3 and Bcl-6 and suppressing FoxP3 expression, thereby impairing Treg-mediated immunoregulation. Pharmacological inhibition of STAT3 by Stattic effectively blocks this pathway and reverses the associated adverse molecular and cellular phenotypes.

## Discussion

T1D is a T cell–mediated autoimmune disease characterized by the selective destruction of pancreatic β cells (24, 25). Dysregulation of CD4^+^ T-cell subsets—manifested as impaired immunosuppressive Treg function accompanied by aberrant activation of pro-inflammatory Th17 and Tfh cells—is widely recognized as a central pathogenic event in disease initiation and progression (26). However, the upstream signaling pathways driving this immune imbalance have not been fully elucidated. By integrating bioinformatic analyses, cell-based functional assays, and animal models, the present study provides multi-level evidence supporting the role of the IL-21/STAT3 signaling axis in T1D. Importantly, the primary value of this work lies in the integration of clinical observations, cellular mechanisms, and *in vivo* disease progression, rather than in the identification of a completely novel signaling pathway. Our findings therefore extend existing knowledge and strengthen the relevance of this axis in T1D immunopathogenesis.

Analysis of publicly available single-cell RNA sequencing datasets provided supportive evidence for the involvement of the IL-21/STAT3 pathway in T1D. We observed enhanced IL-21 signaling activity in peripheral immune cells from patients with T1D, particularly within Tfh and related CD4^+^ T-cell subsets. Our findings are consistent with previous reports (27, 28). IL-21 is well established as a pleiotropic cytokine with critical roles in both humoral and cellular immunity (29). In NOD mouse models, genetic deficiency of IL-21 or its receptor substantially delays or prevents diabetes onset (30), and genetic association studies have identified variants in the IL-21 locus that confer increased susceptibility to human T1D (31). Our single-cell–level analyses refine this understanding by localizing IL-21 pathway activation within discrete immune-cell subsets, suggesting the existence of a self-amplifying inflammatory loop driven by IL-21–producing Tfh cells. These cells may amplify their own responses as well as those of other CD4^+^ T-cell populations through autocrine and paracrine mechanisms. This interpretation is consistent with the observations of McGuire et al., who emphasized the critical role of IL-21 in autoimmune and allogeneic islet responses (32). Thus, our bioinformatic findings bridge population genetics, animal models, and clinical single-cell atlases, supporting IL-21 as a potentially important pathogenic mediator in T1D.

Mechanistically, our study supports an established regulatory framework through which IL-21 regulates CD4^+^ T-cell fate decisions predominantly via STAT3 signaling. We demonstrate that IL-21 induces rapid phosphorylation of STAT3 and nuclear translocation in CD4^+^ T cells. STAT3, a shared signaling mediator among cytokines of the IL-6 family, exerts distinct effects on different T-cell subsets following activation (33, 34). On the pro-inflammatory side, our data show that IL-21 markedly promotes Th17 and Tfh differentiation. This is supported by extensive literature demonstrating that STAT3 directly activates the transcription of RORγt, the master regulator of Th17 differentiation (35, 36), and regulates Tfh cell development and maturation by controlling the expression of Bcl-6 (37, 38). Edelmann et al. further reported that T cell–specific deletion of STAT3 severely impairs Tfh cell generation (39). Collectively, our findings reinforce the IL-21–STAT3–Bcl-6 axis as a canonical and potent driver of Th17/Tfh differentiation.

Conversely, we found that IL-21/STAT3 signaling exerts a pronounced inhibitory effect on Treg cells. IL-21 stimulation resulted in reduced Treg frequency, downregulation of the master transcription factor FoxP3 and the functional molecule CTLA-4, and impaired secretion of immunosuppressive cytokines. At first glance, this appears paradoxical, as some studies have suggested that STAT3 signaling is required for Treg stability and FoxP3 maintenance (40, 41). However, FoxP3 expression is primarily regulated by STAT5, and excessive STAT3 activation may competitively interfere with STAT5 signaling and suppress Foxp3 expression through epigenetic and transcriptional mechanisms (42, 43). In addition, IL-6–induced STAT3 activation suppresses Treg function under inflammatory conditions (44, 45). Although IL-6 exhibited relatively strong effects on T-cell phenotypes in our experimental system, this study focused on IL-21 due to its more specific relevance to Tfh-mediated immune responses and autoimmune diabetes. IL-21 is a key cytokine produced by Tfh cells and has been directly implicated in T1D pathogenesis, particularly in NOD models (28, 46, 47). In contrast, IL-6 functions as a broader inflammatory cytokine across multiple immune contexts (48). Therefore, IL-21 was selected to better reflect disease-relevant immune regulation in T1D. Our study extends this paradigm by identifying IL-21 as another STAT3 activator capable of negatively regulating Treg-associated phenotypes. These findings provide a potential mechanistic explanation for Treg dysfunction observed in the IL-21–enriched inflammatory milieu of T1D and highlight the IL-21/STAT3 axis as a molecular link between inflammatory signaling and impaired immune tolerance.

Through application of the STAT3-specific pharmacological inhibitor Stattic (49, 50), we further established the central role of STAT3 in IL-21 signaling and validated its feasibility as a therapeutic target. In both Treg monoculture and Treg–Teff co-culture systems, Stattic not only blocked downstream molecular changes but also functionally reversed IL-21–induced Treg impairment and pro-inflammatory subset expansion. However, the current co-culture system did not include a condition in which Teff cells were stimulated with IL-21 alone, and thus the relative contributions of direct effects on Teff cells versus indirect effects mediated through Treg dysfunction cannot be fully distinguished. Further studies are needed to clarify these mechanisms. Taken together, these findings support a key role of STAT3 in IL-21–mediated immune dysregulation. This finding may have translational implications. Recent clinical studies targeting IL-21 have demonstrated therapeutic potential in autoimmune diseases (28). Furthermore, our *in vivo* experiments in NOD mice revealed that disease progression was accompanied by increased splenic STAT3 phosphorylation and T-cell imbalance, whereas Stattic intervention concurrently improved glycemic control, pancreatic pathology, splenic immune homeostasis, and systemic cytokine profiles. These observations are consistent with reports showing that STAT3 targeting ameliorates disease manifestations in lupus mouse models (51, 52). Notably, our study provides integrated evidence for the potential multi-level protective effects of STAT3 inhibition, ranging from immune regulation to endocrine organ preservation in a spontaneous T1D model. In addition, the present *in vivo* analyses primarily focused on systemic immune responses and splenic T-cell profiles. Direct evaluation of CD4^+^T-cell signaling and STAT3 activation within pancreatic tissue—the primary site of autoimmune destruction—was not performed, which may limit the mechanistic interpretation at the tissue level.

This work has several limitations that should be taken into account. First, the mechanistic *in vitro* experiments were conducted using CD4^+^ T cells derived from healthy donors rather than pediatric T1D patients. Therefore, the intrinsic activation of STAT3, responsiveness to IL-21, and stability of FoxP3 in patient-derived Tregs remain to be directly validated. Although our single-cell RNA sequencing analysis revealed enhanced IL-21/STAT3 pathway activity in T cells from pediatric T1D patients, the dataset was limited in size, and inter-individual heterogeneity as well as correlations with clinical parameters were not systematically explored, which may influence immune phenotypes and limit clinical interpretation. Moreover, the suppressive capacity of Tregs isolated from treated mice was not assessed, leaving the extent of *in vivo* functional restoration by STAT3 inhibition unclear. Larger patient cohorts and direct validation in patient-derived T cells will be required to strengthen the clinical relevance of these findings. Second, although the specificity of Stattic has been widely validated, off-target actions cannot be fully ruled out. Subsequent investigations utilizing genetic approaches, such as conditional STAT3 deletion models, would yield more conclusive evidence. Third, while our investigation primarily focused on T-cell subsets, the IL-21/STAT3 pathway also affects other immune compartments, such as B cells and macrophages. The contributions of these cells to T1D pathogenesis and their interactions with T cells warrant further exploration. Finally, although the NOD mouse model closely recapitulates many features of human T1D, species-specific differences remain, and the translational potential of these findings will require additional preclinical and clinical validation.

## Conclusion

In conclusion, this study identifies the IL-21/STAT3 signaling pathway as a potentially relevant molecular axis associated with immune dysregulation in T1D. Aberrant activation of this pathway may contribute to Treg functional impairment and the expansion of Th17 and Tfh cells, thereby promoting autoimmune β-cell damage. Pharmacological inhibition of STAT3 partially restores immune balance under experimental conditions, highlighting its potential as a therapeutic target. These findings provide mechanistic insights into T1D immunopathogenesis and support further investigation of the IL-21/STAT3 axis as a candidate immunomodulatory strategy.

## Data Availability

The datasets presented in this study can be found in online repositories. The names of the repository/repositories and accession number(s) can be found below: https://www.ncbi.nlm.nih.gov/genbank/, GSE221297.

## Supplementary material

Supplementary Figure 1 Schematic diagram of the experimental design and treatment timeline in the NOD mouse model.
