# Supplementary figures and images for: Aberrant IL-21/STAT3 signaling disrupts regulatory T cell function and CD4+ T cell homeostasis in children with type 1 diabetes
Source: Front Immunol. 2026 May 18;17:1791993. doi: 10.3389/fimmu.2026.1791993 (PMC13223116; doi:10.3389/fimmu.2026.1791993)

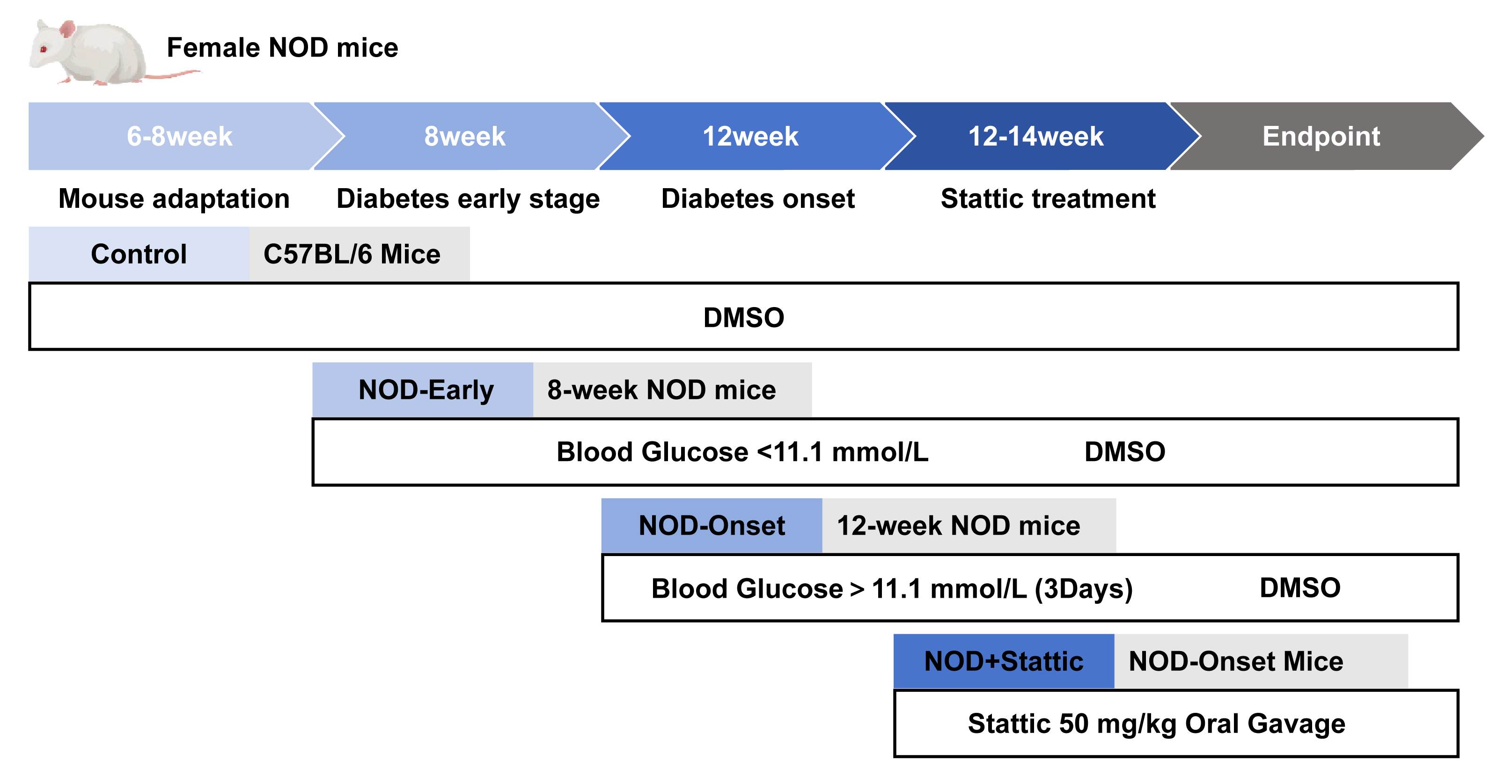

Supplement: Supplementary Figure 1 — Schematic diagram of the experimental design and treatment timeline in the NOD mouse model. [file Image1.tif]
